# Antineoplastic Drug Synergy of Artesunate with Navitoclax in Models of High-Grade Serous Ovarian Cancer

**DOI:** 10.3390/cancers16071321

**Published:** 2024-03-28

**Authors:** J. Robert McCorkle, Rebecca Ahn, Connie D. Cao, Kristen S. Hill, Charles S. Dietrich, Jill M. Kolesar

**Affiliations:** 1 Markey Cancer Center, University of Kentucky, Lexington, KY 40536, USA; rob.mccorkle@uky.edu (J.R.M.); kristen.hill@uky.edu (K.S.H.); charles.dietrich@uky.edu (C.S.D.); 2 University of Kentucky College of Medicine, Lexington, KY 40536, USA; rebecca.ahn@uky.edu; 3 Division of Gynecologic Oncology, Department of Obstetrics and Gynecology, University of Kentucky, Lexington, KY 40536, USA; connie.cao@uky.edu; 4 Department of Pharmacy Practice and Science, University of Kentucky College of Pharmacy, Lexington, KY 40536, USA; 5 Department of Clinical Research, University of Kentucky Markey Cancer Center, Lexington, KY 40536, USA

**Keywords:** artesunate, navitoclax, drug synergy, ovarian cancer

## Abstract

**Simple Summary:**

Modern ovarian cancer treatment has not substantially improved outcomes, and superior therapeutic strategies are needed. The aim of this study was to evaluate the efficacy of artesunate and navitoclax drug combination in ovarian cancer. We determined the combination of these two drugs was extraordinarily effective in multiple models of ovarian cancer, in vitro, inhibiting cancer cell proliferation more than expected based on single-agent activities. Unfortunately, we were unable to validate these findings using a mouse model of metastatic ovarian cancer. These data provide valuable information regarding the potential utility and challenges associated with the artesunate/navitoclax drug combination for ovarian cancer therapy.

**Abstract:**

Artesunate belongs to a class of medications derived from the sweet wormwood plant (*Artemisia annua*) known as artemisinins. Artesunate has traditionally been used as a frontline treatment for severe malaria but has also demonstrated antineoplastic activity against various malignancies, including ovarian cancer. Data suggest that artesunate exacerbates cellular oxidative stress, triggering apoptosis. In the current study, we investigated the ability of navitoclax, an inhibitor of the antiapoptotic Bcl-2 protein family, to enhance artesunate efficacy in ovarian cancer cells. Artesunate and navitoclax both demonstrated antiproliferative effects on 2D and 3D ovarian cancer cell models as single agents. Upon combination of navitoclax with artesunate, antineoplastic drug synergy was also observed in each of the 2D cell lines and ovarian tumor organoid models tested. Further investigation of this drug combination using intraperitoneal CAOV3 xenograft models in BALB/scid mice showed that the artesunate/navitoclax doublet was superior to single-agent artesunate and vehicle control treatment. However, it did not outperform single-agent navitoclax. With optimization, this drug combination could provide a new therapeutic option for ovarian cancer and warrants further preclinical investigation.

## 1. Introduction

Ovarian cancer is the deadliest gynecologic malignancy, accounting for an estimated 13,270 deaths in the United States in 2023 [1]. Although ovarian cancer makes up only 1% of all new cancer cases, the estimated annual death rate from ovarian cancer is 2.2% [1]. With death rates higher than occurrences and five-year overall survival rates near 50%, it is crucial to find improved therapeutic strategies for ovarian cancer. 

First-line therapy for ovarian cancer includes cytoreduction surgery and adjuvant chemotherapy with a taxane and platinum doublet, a regimen that has been largely unchanged for 30 years [2]. More recently, select patients have received maintenance therapy with either bevacizumab and/or a PARP inhibitor, the latter of particular benefit for *BRCA*-mutated ovarian cancers, which account for approximately 15% of all cases [3,4,5,6]. However, more than 70% of patients have advanced-stage cancer at diagnosis [7], and up to 80% of these patients develop recurrence and ultimately die of their disease [8], highlighting the need for novel treatments. 

Artesunate is a semisynthetic derivative of artemisinin, an extract from the sweet wormwood plant, *Artemisia annua* [9], which has been used as an antipyretic in Chinese herbal medicine for over 2000 years [8]. Artesunate is currently used to treat malaria but has also demonstrated antineoplastic activity across a broad spectrum of cancer cell lines, including ovarian cancer [8,10,11]. The primary mechanism of anticancer activity for artesunate is thought to be generation of reactive oxygen species (ROS), leading to oxidative damage of DNA and proteins resulting in apoptosis [11,12,13,14,15,16,17]. Forty years of use as malaria therapy and cancer clinical trials have demonstrated that artesunate is also well tolerated [12,18]. 

Navitoclax is a small-molecule inhibitor of the Bcl-2 family (i.e., BCL2, BCL2L1, BCL2L2) of antiapoptotic proteins with demonstrated activity in preclinical ovarian cancer models, including an ex vivo model of 25 high-grade serous ovarian cancers [19]. Based on promising preclinical data, navitoclax as a single agent was assessed in a phase 2 trial that included 45 heavily pretreated women with high-grade serous, platinum-resistant, or refractory ovarian cancer. In this trial, navitoclax showed limited activity, with only one patient achieving a partial response, although adverse effects were acceptable, with thrombocytopenia the major toxicity [19,20,21]. In other cancers, navitoclax in combination with chemotherapy has been effective, although limited by toxicity [22,23].

Given that artesunate induces apoptosis, assessing potential drug synergy utilizing an inhibitor of Bcl-2 proteins is a plausible route of investigation, which could also improve the single-agent activity of navitoclax. Moreover, the acceptable toxicity profiles of both drugs make them attractive candidates for clinical investigation. Although artemisinins have been preclinically evaluated in combination with Bcl-2 inhibitors in leukemia and non-small-cell lung cancer cells [24,25,26], the use of artesunate with navitoclax has not been investigated in ovarian cancer. We examined antitumor efficacy of this regimen using 2D and 3D human ovarian tumor models, both in vitro and in vivo.

## 2. Materials and Methods

### 2.1. 2D Cell Culture

Four high-grade serous ovarian carcinoma cell lines, OVCAR3, UWB1.289, CAOV3, and OV-90, were purchased from ATCC. Cells were cultured under constant conditions of 37 °C and 5% CO_2_ and passaged 2 to 3 times per week. OVCAR3 cells were grown in RPMI-1640 base medium with 20% fetal bovine serum and 0.01 mg/mL bovine insulin; UWB1.289 cells were grown in a 1:1 ratio of RPMI-1640 and Mammary Epithelial Growth Medium (MEGM) supplemented with 3% fetal bovine serum; CAOV3 cells were grown in DMEM with 10% fetal bovine serum; and OV-90 cells were grown in a 1:1 mixture of MCDB 105 and Medium 199 supplemented with 15% fetal bovine serum.

### 2.2. 2D Cell Viability Assay

Cells (3 × 10^3^) were seeded in 96-well white-walled microplates in 100 μL growth media and allowed to attach for 24 h at 37 °C, 5% CO_2_. Growth media were then removed and replaced with drug containing media or blank media for negative controls. Each cell line was grown in the presence of serially diluted navitoclax or artesunate for 72 h. Drug concentrations of artesunate ranged from 0.05 μM to 100 μM (12 concentrations; 4-fold dilutions); navitoclax concentrations ranged from 0.01 μM to 25 μM (12 concentrations; 2-fold dilutions). Following treatment, cell viability (%) was measured using CellTiter-Glo 2.0 Cell Viability Assay (Promega, Chūō, Tokyo) with a Varioskan LUX multimode microplate reader (ThermoFisher Scientific, Waltham, MA, USA) and normalized to untreated control cells (drug-treated luminescence/mean untreated luminescence × 100). Four-parameter log-logistic nonlinear models were used to determine the IC_50_ for each drug in each cell line using package *drc* [27] within R statistical software (version 4.1.1).

### 2.3. 2D Drug Combination/Synergy Analysis

Cell viability was measured following 72 h of treatment using CellTiter-Glo 2.0 cell proliferation assays, as described above. Artesunate and navitoclax were tested alone and in combination in a 6 × 6 full factorial synergy study design with at least 3 independent experiments per cell line. The tested concentrations of artesunate ranged from 0 μM to 200 μM and navitoclax ranged from 0 μM to 40 μM. Drug synergy was analyzed using the *synergyfinder* package within R statistical software (version 4.1.1) [28]. Synergy scoring was implemented using the Loewe additivity model [29].

### 2.4. 3D Tumor Organoid Culture

Tumor organoid cell line, UK1254, was established from a pathologically confirmed advanced-stage epithelial ovarian cancer, originating in the ovary of a 49-year-old patient treated at the Markey Cancer Center [8,30]. Organoids were cultured under constant conditions of 37 °C and 5% CO_2_, passaged once a week with 2 media changes per week. Cells were grown in Corning^®^ Matrigel^®^ Growth Factor Reduced Basement Membrane Matrix. Base medium used was Advanced DMEM/F12 with additives of R-Spondin-1(RSPO-1) conditioned media from HEK293 RSPO-1 expressing cells (20% vol/vol), Wnt-3A (50 ng/mL), FGF10 (100 ng/mL), Noggin (100 ng/mL), EGF (10 ng/mL), A83-01 (500 nM), Y-27632 (9 uM), B27 supplement (2% vol/vol), N2 supplement (1% vol/vol), nicotinamide (1 mM), Glutamax (2mM), HEPES (10 mM), and primocin (50 ug/mL).

### 2.5. Organoid Viability Assay

UK1254 organoids were grown in the presence of serially diluted navitoclax or artesunate for 72 h. Cell viability was measured using CellTiter-Glo 3D Cell Viability Assay (Promega). Cell viability was assessed using high-throughput imaging and compared to untreated control cells to determine response at each drug concentration. Four-parameter log-logistic nonlinear regression models were used to determine the IC_50_ for each drug. A total of 6 concentrations ranging from 0 μM to 16 μM (10-fold dilutions) were tested for artesunate, and 6 concentrations ranging from 0 μM to 12.5 μM (2-fold dilutions) were tested for navitoclax.

### 2.6. Drug Combination/Synergy Analysis in Organoids 

Dose–response data were produced using cell proliferation assays, as described above. Pairs of drugs were tested in combination with each of five serially diluted concentrations in a full factorial synergy study design, with a sixth concentration serving as the control. The percentage of viable cells relative to untreated control cells was assayed for drug–drug interactions using the Bioconductor *synergyfinder* package within R statistical software (version 4.1.1) [28,31]. Synergy scoring was implemented using the Loewe additivity [29], Bliss independence [32], highest single agent (HSA) [33], and zero interaction potency (ZIP) models [34]. Synergy scores = 0 indicated additivity; scores > 0 indicated synergy; scores < 0 represented antagonism. The concentration range for artesunate was 0 μM to 50 μM (4-fold dilutions) and 0 μM to 12.5 μM for navitoclax (2-fold dilutions).

### 2.7. Apoptosis Assay

CAOV3 and OVCAR3 ovarian cancer cells (4 × 10^5^) were plated in 60 mm dishes and incubated at 37 °C, 5% CO_2_ for 24 h. Cells were then treated with 10 μM navitoclax, 25 μM artesunate (OVCAR3), or 50 μM artesunate (CAOV3) for 48 h. To assess drug combination, cells were also treated with 10 μM artesunate and/or 10 μM navitoclax for 48 h. Media were removed and cells were washed with D-PBS and detached using Accutase cell detachment solution (BD Biosciences). Cells were collected, centrifuged for 5 min at 300× *g*, washed twice with ice-cold D-PBS, then resuspended in 1X Annexin V Binding Buffer (BD Biosciences). Cells were passed through a 70 μm mesh filter then labeled for 15 min with FITC-Annexin V and propidium iodide using the FITC Annexin V Apoptosis Detection Kit I (BD Biosciences). Cells were subsequently analyzed by flow cytometry.

### 2.8. In Vivo Xenograft Mouse Models

Animal studies were approved by the University of Kentucky Institutional Animal Care and Use Committee. BALB/c scid (CBySmn.Cg-*Prkdc^scid^*/J) mice were purchased from The Jackson Laboratory and maintained in barrier cages under pathogen-free conditions. 

Luciferase and GFP-positive CAOV3 cells (CAOV3-luc) were generated by lentiviral transduction using pLL-CMV-rFLuc-GFP-mPGK-Puro Lenti-Labeler lentivirus (System Biosciences) at a multiplicity of infection (MOI) of 15. Transduced cells were selected in CAOV3 culture media containing 1 μg/mL puromycin for 2 weeks. Luciferase/GFP-positive cells were further enriched by FACS, collecting the 25% of cells with highest GFP expression. Sorted cells were maintained in 1 μg/mL puromycin.

Six-week old female BALB/c scid mice were injected intraperitoneally with 1.25 × 10^7^ CAOV3-luc cells. To monitor cell engraftment, mice were administered 150 mg/kg IVISbrite D-luciferin potassium salt (PerkinElmer) by intraperitoneal injection. Mice were anesthetized using 3% isoflurane inhalation and imaged once weekly using a Lago optical imaging system (Spectral Instruments) to assess peak whole-body bioluminescence 5–15 min post-injection. Bioluminescence was quantified using Aura image analysis software (version 4.0.7) (Spectral Instruments).

Evaluation of treatment efficacy was conducted using groups of eight tumor-bearing mice randomly assigned to receive vehicle, artesunate alone, navitoclax alone, or artesunate in combination with navitoclax. Groups had equivalent mean tumor burden based on bioluminescence measurement at the start of treatment. Artesunate and navitoclax were purchased from MedChemExpress and prepared in 10% DMSO, 40% PEG-300, 5% Tween-80, and 45% saline. Treatment was given once daily for 5 days (Monday–Friday) for 8 weeks. Navitoclax was given at 50 mg/kg. Artesunate was administered at 50 mg/kg for the first 4 weeks of treatment, then was increased to 100 mg/kg for the subsequent 4 weeks. Bioluminescence imaging was conducted weekly during treatment as described. 

### 2.9. Statistical Analysis

Statistical analyses were performed using R (version 4.0.1) or GraphPad Prism. Statistical significance was designated as * *p* < 0.05, ** *p* < 0.01, and *** *p* < 0.001.

## 3. Results

### 3.1. Single-Agent Sensitivity Analyses

**Figure 1:**
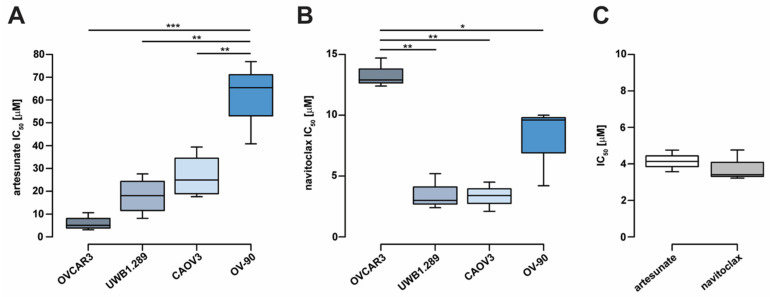
Ovarian cancer cell line sensitivity to single-agent artesunate and navitoclax treatment. (**A**) Ovarian cancer cell line IC_50_ values estimated following 72 h of artesunate treatment or (**B**) navitoclax treatment. (**C**) IC50 values for artesunate and navitoclax similarly determined in novel ovarian tumor organoid line, UK1254. Box and whiskers depict interquartile ranges and minimum/maximum values, respectively, with median values shown as lines within boxes. Statistical analyses were conducted with one-way analysis of variance and Tukey’s multiple comparisons post hoc tests (* *p* < 0.05; ** *p* < 0.01; *** *p* < 0.001).

Relative de novo sensitivity to artesunate was evaluated in a panel of human high-grade serous ovarian cancer cell lines including CAOV3, OVCAR3, OV-90, and BRCA1-null UWB1.289 cells. Due to time-dependent effects of artesunate on ovarian cancer cell proliferation, we chose to assess cell viability following 72 h of treatment, consistent with previous studies [8,11]. Cell viability assays were performed and IC_50_ values were calculated. Relative artesunate sensitivity differed by approximately 10-fold across cell lines (Figure 1A). Mean artesunate IC_50_ for OVCAR3, UWB1.289, CAOV3, and OV-90 measured 5.95 μM (standard deviation (SD) = 3.25), 17.95 μM (SD = 8.34), 26.73 μM (SD = 9.89), and 61.00 μM (SD = 18.40), respectively. Artesunate IC_50_ was significantly higher in OV-90 cells compared to CAOV3, UWB1.289, and OVCAR3 cells (one-way ANOVA *p* = 2.0 × 10^−4^; Tukey’s multiple comparison *p* < 0.01). Previous studies have shown that artesunate exposure induces apoptosis in cancer cells [10,15,17,35,36,37]; therefore, we aimed to determine if inhibiting antiapoptotic Bcl-2 family proteins could enhance artesunate efficacy in ovarian cancer cells. Navitoclax belongs to a class of BH3-only mimetic small molecule inhibitors of Bcl-2, Bcl-xL, and Bcl-w. Sensitivity to navitoclax was evaluated in the ovarian cancer cell line panel where the mean IC_50_ values were 3.33 μM (SD = 1.20), 3.53 μM (SD = 1.47), 7.93 μM (SD = 3.24), and 13.33 μM (SD = 1.21) for CAOV3, UWB1.289, OV-90, and OVCAR3, respectively (Figure 1B). Navitoclax IC_50_ was significantly higher in OVCAR3 cells compared to UWB1.289, CAOV3, and OV-90 cells (one-way ANOVA *p* = 7.8 × 10^−4^; Tukey’s *p* < 0.05). 

The novel epithelial ovarian cancer organoid line, UK1254 [8,30], was also assessed for artesunate and navitoclax sensitivity (Figure 1C). The mean artesunate IC_50_ for UK1254 organoids was 4.15 μM (SD = 0.59) and the mean navitoclax IC_50_ was 3.80 μM (SD = 0.84), comparable to values observed in the 2D cell lines.

### 3.2. Artesunate in Combination with Navitoclax

**Figure 2:**
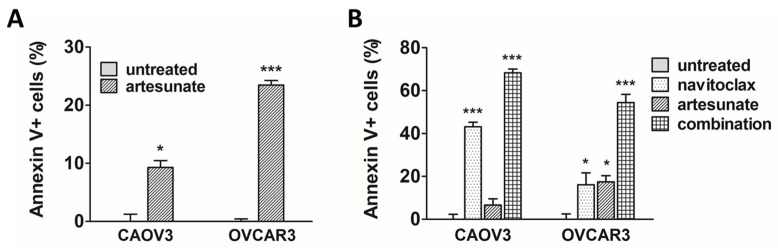
Apoptosis induction following treatment with artesunate and navitoclax. (**A**) CAOV3 and OVCAR3 cells were treated for 48 h with artesunate, and apoptosis was analyzed. Mean percentage of apoptotic cells (error bars = SEM) depicted relative to untreated controls. Statistical analyses conducted with unpaired, two-tailed t-tests. (**B**) Ovarian cancer cells treated for 48 h with artesunate and navitoclax, alone and in combination, were analyzed for apoptosis induction. Mean percentage of apoptotic cells (error bars = SEM) depicted relative to untreated controls. Statistical analyses conducted with one-way analysis of variance and Dunnett’s multiple comparisons tests (vs. untreated control) (* *p* < 0.05; *** *p* < 0.001).

To confirm that the observed artesunate-mediated loss of cell viability was indeed due to apoptosis in our model systems, artesunate-sensitive CAOV3 and OVCAR3 cells were treated with 50 μM and 25 μM artesunate for 48 h, respectively. Apoptosis was measured using FITC-Annexin V and propidium iodide staining with flow cytometry analysis. After normalizing to untreated controls, a 9.3% (SD = 1.4) increase in apoptosis was observed in CAOV3 cells while apoptosis was induced in 23.5% (SD = 1.3) of OVCAR3 cells (Figure 2A) by artesunate. In CAOV3 (*p* < 0.05) and OVCAR3 cells (*p* < 0.001), rates of apoptosis were significantly higher than basal rates observed in untreated cells. Next, apoptosis was examined following combination treatment using moderately cytotoxic single-agent concentrations of artesunate and navitoclax at a 1:1 molar ratio. CAOV3 and OVCAR3 cells were treated with 10 μM each of artesunate and navitoclax, alone and in combination, for 48 h. As shown in Figure 2B, 10 μM navitoclax induced apoptosis in 43.1% (SD = 2.3) of CAOV3 (*p* < 0.05) and 16.1% (SD = 9.2) of OVCAR3 cells (*p* < 0.05). Artesunate alone triggered apoptosis in 6.7% (SD = 4.2) and 17.4% (SD = 3.9) of CAOV3 (*p* > 0.05) and OVCAR3 cells (*p* < 0.05), respectively. Upon combination, apoptosis increased to 68.3% (SD = 1.6) of CAOV3 cells (*p* < 0.001) and 54.4% (SD = 6.0) of OVCAR3 cells (*p* < 0.001).

**Figure 3:**
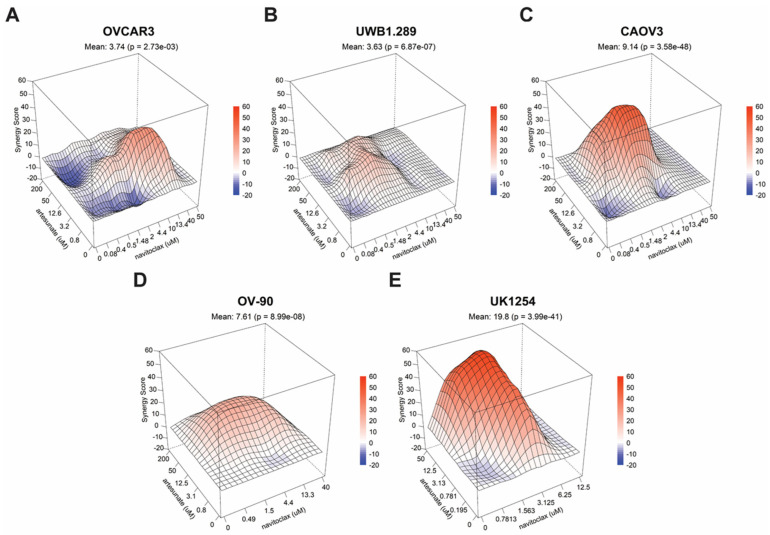
Drug synergy in ovarian cancer cells treated with artesunate with navitoclax. 3D surface plots of Loewe synergy scores across a range of combinations of artesunate and navitoclax drug concentrations in (**A**) OVCAR3, (**B**) UWB1.289, (**C**) CAOV3, (**D**) OV-90, and (**E**) UK1254 cells. Mean synergy scores for all values in the matrix and associated p-values are shown.

A formal analysis of drug synergy in the full ovarian cancer cell line panel followed. After 72 h of treatment, cytotoxic drug synergy was observed at clinically relevant concentrations in all ovarian cancer cell lines tested (Figure 3A–D), including artesunate-resistant OV-90 cells. Using the Loewe additivity model [29] to evaluate synergy, we found that the combination significantly outperformed the null model of noninteraction in CAOV3 (*p* = 3.58 × 10^−48^), OVCAR3 (*p* = 2.73 × 10^−3^), UWB1.289 (*p* = 6.87 × 10^−7^) and OV-90 cells (*p* = 8.99 × 10^−8^). Moreover, potent drug synergy was seen in the UK1254 organoid line (*p* = 3.99 × 10^−41^) (Figure 3E). To ensure that the apparent synergism for artesunate and navitoclax was not model dependent, we analyzed our data using Bliss independence, zero interaction potency (ZIP), and highest single agent (HSA) models, as well (Table S1). Artesunate and navitoclax was deemed synergistic under each model for all cell lines tested, with the exception of UWB1.289 cells, where the combination was classified as additive in two of four models.

### 3.3. Artesunate with Navitoclax in Ovarian Cancer Xenografts

Drug synergy observed in vitro prompted further investigation of the artesunate plus navitoclax combination in vivo using orthotopic mouse xenografts. CAOV3-luc cells were generated to monitor intraperitoneal tumor growth in mice using whole-body bioluminescence imaging. Briefly, CAOV3 cells were labeled with a green fluorescent protein (GFP) and firefly luciferase (rFLuc) coexpression lentiviral vector, and cells with stable genomic integration were selected using FACS and puromycin (CAOV3-luc). Intraperitoneal tumor xenografts were established in 6-week-old female BALB/c scid mice and engraftment was monitored weekly with bioluminescence imaging. 

Five weeks post-implantation, engraftment was established and 32 CAOV3-luc tumor-bearing mice were randomized into four treatment groups of equivalent numbers, weights, and tumor burdens: vehicle control, artesunate alone, navitoclax alone, and combination. A dosing regimen was developed based on previous studies demonstrating efficacy with minimal toxicities in mouse xenograft models [38,39,40,41,42,43,44,45]. Treatment was administered via oral gavage 5 days per week for 8 weeks with navitoclax given at 50 mg/kg. Artesunate was given at 50 mg/kg for the first 4 weeks then escalated to 100 mg/kg for weeks 5–8 due to lack of apparent efficacy at the lower dose. Treatment was well tolerated, with minimal drug-related toxicities observed. There were three instances of epistaxis in two mice in the navitoclax alone group, which resolved without intervention. 

**Figure 4:**
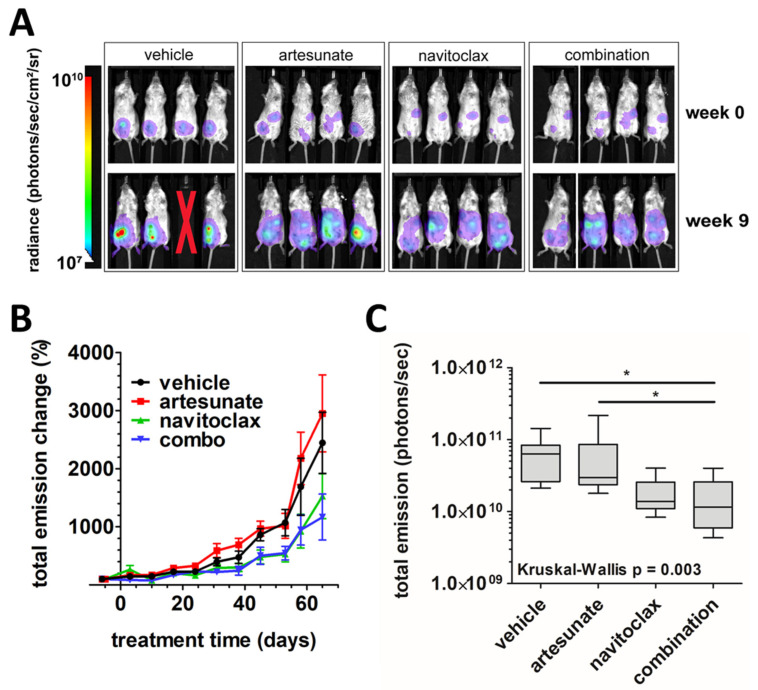
In vivo analysis of artesunate and navitoclax, alone and in combination, in ovarian cancer xenografts. (**A**) Representative images of tumor xenograft bioluminescence in mice treated with vehicle, artesunate alone, navitoclax alone, or combination at beginning (week 0) and end (week 9) of treatment. Red X indicates a mouse that died during treatment. (**B**) The mean percent change (error bars = SEM) in total emissions from the week prior to treatment to 1 week post-treatment plotted versus time for vehicle (black), artesunate alone (red), navitoclax alone (green), and combination (blue) treatment groups. (**C**) Box and whisker plots of total emissions from tumor xenograft bioluminescence 1 week post-treatment. Lines indicate median values within treatment groups. Kruskal–Wallis test (*p* = 0.003) and Dunn’s multiple comparison tests were used for statistical analysis (* *p* < 0.05).

Figure 4A shows representative images of tumor xenograft bioluminescence among each treatment group at the beginning and end of treatment. Quantitation of the change in total emissions over time relative to pretreatment levels (mean % ± SEM) is summarized in Figure 4B. A higher rate of tumor progression became apparent in the vehicle and artesunate alone groups compared to navitoclax alone and combination treatment groups during weeks 4–8 of treatment. During treatment, one mouse within the vehicle control group died due to apparent isoflurane overexposure on day 10 and was censored from the study. Another vehicle-treated mouse succumbed to disease during week 6 of treatment. The remaining 30 mice survived the entirety of the study. One week following conclusion of treatment, bioluminescence was measured and median total emissions were significantly lower in the combination treatment group compared to the vehicle and single-agent artesunate-treated mice (6.30 × 10^10^ photons/second (vehicle) versus 2.96 × 10^10^ p/s (artesunate) versus 1.15 × 10^10^ p/s (combination); *p* < 0.05; Figure 4C). No significant difference was seen in median bioluminescence between single-agent navitoclax (1.38 × 10^10^ p/s) and other treatment groups or between vehicle and artesunate alone groups. 

## 4. Discussion

Ovarian cancer remains the most lethal gynecologic malignancy, yet development of new treatment modalities has been limited. Contemporary frontline treatment consisting of a platinum/taxane doublet has not significantly changed in nearly three decades, despite limited improvement in outcomes. Clearly, identifying better therapeutic strategies for ovarian cancer is of the utmost importance.

Artesunate is a water-soluble, orally bioavailable, semisynthetic derivative of artemisinin that has demonstrated cytotoxic activity against a variety of cancer cells [10,18]. Our present and prior work shows that artesunate inhibits cell proliferation and induces apoptosis in human ovarian cancer cell lines and tumor organoids [8]. Artemisinins have also shown the ability to enhance the cytotoxic effects of platinum agents in ovarian cancer cells [46,47,48]. While most evidence of anticancer efficacy comes from preclinical studies, artesunate has exhibited clinical activity in early-phase clinical trials [18,49,50]. Moreover, artemisinins, including artesunate, are well tolerated, having been widely used throughout the world as frontline treatment for malaria without reports of any serious adverse events [51]. Taken together, these data support further clinical investigation of artesunate for cancer treatment.

Navitoclax belongs to a class of drugs known as BH3 mimetics, which augment the induction of apoptosis [52]. Upon binding the BH3 domain of Bcl-2 proteins, navitoclax disrupts the sequestration of the proapoptotic BIM protein, causing its release and eventually apoptosis. Clinical evaluation of single-agent navitoclax has been met with limited success, with activity falling short of expectations and significant dose-limiting but manageable thrombocytopenia. In a phase II study with small-cell lung cancer patients (SCLC), Rudin et al. reported a partial response in 2.6% of patients, stable disease in 23.1% of patients, and progression in 41% of patients [53]. Furthermore, grade III–IV thrombocytopenia occurred in 41% of those on study. The MONAVI-GINECO study evaluated navitoclax monotherapy in recurrent epithelial ovarian cancer [19]. Like outcomes in SCLC, a partial response was observed in 2.2% of patients, stable disease in 32.6% of patients, and progressive disease in 65.2% of patients. Grade III–IV thrombocytopenia occurred in 26% of patients, resulting in discontinuation of treatment in 25% of affected individuals. Consistent failure of navitoclax monotherapy to produce acceptable outcomes has prompted a shift in focus to combination treatment strategies which could enhance efficacy and avoid dose-limiting toxicities.

Both chemotherapy and targeted agents, including rituximab, dasatinib, vemurafenib, vorinostat, and tamoxifen, have demonstrated enhanced efficacy when combined with navitoclax in solid tumors and hematologic cancers [54,55,56,57,58,59]. Navitoclax enhanced the in vitro activity of carboplatin and paclitaxel in 2D and 3D ovarian cancer cell cultures and was synergistic with rucaparib in HRD ovarian cancer cells [60,61]. Our own work shows that navitoclax in combination with artesunate is synergistic in 2D human ovarian cancer cell lines as well as in a novel high-grade serous ovarian cancer organoid model. 

Artemisinins combined with navitoclax have demonstrated enhanced benefit over monotherapy in several cancer models, including leukemia and non-small-cell lung cancer [24,26,62,63]. We aimed to show that artesunate with navitoclax is a promising treatment strategy for metastatic ovarian cancer. As presented in Figure 3, in vitro drug synergy was significant and consistent across various ovarian cancer cell models. This included an artesunate-resistant cell line, OV-90, a *BRCA1*-mutant cell line, UWB1.289, and a patient-derived tumor organoid line (UK1254). Unfortunately, the in vitro results did not translate to animal models. While the in vivo study did demonstrate that combination therapy was superior to the vehicle control and artesunate monotherapy, it did not outperform single-agent navitoclax. This suggests that navitoclax is driving the observed combination treatment benefit. 

The failure of combination treatment to outperform single-agent navitoclax was unexpected; however, there are several possible explanations. First, the route of drug administration may not have been optimal to maximize the bioavailability of artesunate. Thus, artesunate concentrations could have been below the threshold needed to achieve drug synergy with navitoclax at the tumor site. In healthy individuals, the absolute bioavailability of oral artesunate was estimated only to be 21.6% [64]. It is possible that navitoclax combination treatment using intravenous artesunate is more effective. Second, the frequency of artesunate dosing may have been insufficient. Reported artesunate half-life estimates in humans indicates a relatively short half-life. A review of the available literature found that nearly all studies estimated artesunate half-life to be less than 1 h following oral treatment [65]. The short elimination half-life was further supported by a reportedly small volume of distribution [64]. Our in vivo study may have required increased frequency of oral artesunate dosing to achieve intratumoral concentrations necessary for navitoclax drug synergy. Lastly, tumors may have been allowed to progress to a point where they were no longer responsive to artesunate treatment. As indicated by the small volume of distribution, artesunate may have limited distribution into tissues that are not well perfused. The peritoneum and omentum are highly vascularized, providing ample opportunity for perfusion of artesunate from the plasma into the peritoneal space. Although we used an orthotopic intraperitoneal model of metastatic ovarian cancer that should have enabled sufficient drug exposure, CAOV3-luc cells were allowed to engraft for >4 weeks prior to treatment. This may have permitted establishment of macro-metastases, hindering penetration of artesunate into larger tumor nodules. An alternative strategy in which combination treatment was initiated much sooner, targeting micro-metastases and circulating tumor cells, may have produced the expected synergistic effect. Consequently, this could suggest that artesunate with navitoclax holds greater future clinical benefit if used as adjuvant treatment in patients that have yet to develop distant metastases. 

## 5. Conclusions

As discussed, navitoclax synergizes with artesunate to kill ovarian cancer cells at clinically achievable concentrations in both artesunate-sensitive and resistant cells, in vitro. This drug combination could help fulfill the urgent need for new therapeutic options in ovarian cancer; however, preclinical optimization is required. Clinically, the lack of consensus for effective doses and method(s) of administration for artesunate hinders its advancement as a practical cancer therapy. However, decades of use for malaria treatment have demonstrated a favorable safety profile for artesunate which may translate to advantageous therapeutic indexes when combined with suitable drugs, like navitoclax. Continued preclinical investigation of this treatment combination is critical for realizing its therapeutic potential in ovarian cancer.

## Data Availability

The data presented in this study are available on request from the corresponding author.

## Supplementary Materials

The following supporting information can be downloaded at: https://www.mdpi.com/article/10.3390/cancers16071321/s1, Table S1: Artesunate and navitoclax synergy scores using various synergy models.
