# Supplementary material for: Antineoplastic Drug Synergy of Artesunate with Navitoclax in Models of High-Grade Serous Ovarian Cancer
Source: Cancers (Basel). 2024 Mar 28;16(7):1321. doi: 10.3390/cancers16071321 (PMC11011058; doi:10.3390/cancers16071321)
Supplement: Supplementary file 1 [file cancers-16-01321-s001.zip › cancers-2924583-supplementary.pdf]

Table S1. Artesunate and navitoclax synergy scores using various synergy models

Cell line	Synergy Models							
	Loewe		Bliss		ZIP		HSA	
	synergy score	p-value	synergy score	p-value	synergy score	p-value	synergy score	p-value
OVCAR3	3.74	2.73×10^{-3}	4.61	1.17×10^{-2}	4.93	5.60×10^{-4}	7.03	1.41×10^{-8}
UWB1.289	3.63	6.87×10^{-7}	0.45	7.09×10^{-1}	0.58	6.00×10^{-1}	6.09	4.74×10^{-9}
CAOV3	9.14	3.58×10^{-48}	8.66	1.36×10^{-20}	9.04	4.46×10^{-24}	11.56	2.76×10^{-69}
OV-90	7.61	8.99×10^{-8}	5.87	2.84×10^{-4}	6.32	8.07×10^{-5}	11.58	1.05×10^{-14}
UK1254	19.8	3.99×10^{-41}	17.17	6.70×10^{-25}	18.04	6.87×10^{-26}	21.33	3.28×10^{-53}
